# Sit-to-stand test as a complementary assessment in patients with cardiovascular disease

**DOI:** 10.15649/cuidarte.4864

**Published:** 2026-05-08

**Authors:** Juan Carlos Avila-Valencia, Jhonatan Betancourt-Peña, Nicol Andrea Saavedra-Orozco, Vicente Benavides-Cordoba

**Affiliations:** 1 Institución Universitaria Escuela Nacional del Deporte. Clínica de Occidente S. A. Cali, Colombia. E-mail: rhcardiopulmonar09@gmail.com Clínica de Occidente S. A. Cali Colombia rhcardiopulmonar09@gmail.com; 2 Institución Universitaria Escuela Nacional del Deporte. Cali, Colombia. E-mail: johnnatanbp@hotmail.com Institución Universitaria Escuela Nacional del Deporte Cali Colombia johnnatanbp@hotmail.com; 3 Institución Universitaria Escuela Nacional del Deporte. Cali, Colombia. E-mail: orozconicolandrea@gmail.com Institución Universitaria Escuela Nacional del Deporte Cali Colombia orozconicolandrea@gmail.com; 4 Universidad del Valle. Cali, Colombia. E-mail: vicente.benavides@correounivalle.edu.co Universidad del Valle Cali Colombia vicente.benavides@correounivalle.edu.co

**Keywords:** Walk Test, Exercise Test, Cardiovascular Diseases, Prueba de Caminata de 6 Minutos, Prueba de Ejercicio, Enfermedades Cardiovasculares, Teste de Caminhada, Teste de Esforço, Doenças Cardiovasculares

## Abstract

**Introduction:**

The 1-Minute Sit to Stand Test (STS1) has gained relevance as a simple, safe, and functional test in patients with cardiovascular disease, especially in settings where the 6-Minute Walk Test (6MWT) is not feasible. Its usefulness in assessing lower-extremity muscle strength makes it a complementary tool of increasing interest that can strengthen functional assessment batteries in this population.

**Objective:**

To assess the correlation between the 6MWT and the STS1 as complementary tools in the functional assessment of patients with cardiovascular disease.

**Materials and Methods:**

Descriptive, correlational, cross-sectional study conducted in 45 patients enrolled in a cardiac rehabilitation program at a quaternary care clinic in Cali, Colombia. The 6MWT and STS1 were administered, assessing physiological variables and physical performance variables. Pearson’s correlation coefficient was used for bivariate analysis.

**Results:**

The mean age was 62.97 ± 12.58 years; 64.4% were women. Coronary artery disease was the most common diagnosis (73.3%). A moderate positive correlation was found between the distance walked in the 6MWT and the number of repetitions in the STS1 (r=0.610; p<0.001). The 6MWT elicited a greater increase in heart rate (final: 112.7 ± 18.1), whereas the STS1 had a greater impact on systolic blood pressure (final SBP: 146.8 ± 27.9; p<0.001).

**Discussion:**

The results are consistent with studies in other populations and support the use of STS1 as a valid functional assessment option.

**Conclusion:**

The STS1 complements the 6MWT in the functional assessment of patients with cardiovascular diseases.

## Resumen

**Introducción:**

El Sit to Stand de 1 Minuto (STS1) ha ganado relevancia como prueba funcional sencilla, segura y aplicable en pacientes con enfermedades cardiovasculares, especialmente en escenarios donde el Test de Caminata de 6 Minutos (TC6M) no es viable. Su utilidad para valorar la fuerza de miembros inferiores lo convierte en una herramienta complementaria de interés creciente, que puede fortalecer las baterías de evaluación funcional en esta población.

**Objetivo:**

Correlacionar los resultados del TC6M con el STS1 como herramientas complementarias en la evaluación funcional de pacientes con enfermedad cardiovascular.

**Materiales y métodos:**

Estudio descriptivo, correlacional y de corte transversal realizado en 45 pacientes que ingresaron a un programa de rehabilitación cardiaca en una clínica de cuarto nivel en Cali, Colombia. Se aplicaron el TC6M y el STS1, evaluando variables fisiológicas y de rendimiento físico. Se utilizó el coeficiente de correlación de Pearson para el análisis bivariado.

**Resultados:**

Edad promedio de 62,97 ± 12,58 años; el 64,4% fueron mujeres. La enfermedad coronaria fue el diagnóstico más común (73,3%). Se encontró una correlación positiva moderada entre la distancia recorrida en el TC6M y el número de repeticiones en el STS1 (r=0,610; p<0,001). El TC6M generó un mayor incremento en la frecuencia cardiaca (final: 112,7 ± 18,1), mientras que el STS1 impactó más sobre la presión arterial sistólica (PAS final: 146,8 ± 27,9; p<0,001).

**Discusión:**

Los resultados coinciden con estudios en otras poblaciones y respaldan el uso del STS1 como opción funcional válida.

**Conclusiones:**

El STS1 complementa al TC6M en la evaluación funcional de pacientes con enfermedades cardiovasculares.

## Resumo

**Introdução:**

O teste Sit to Stand de 1 Minuto (STS1) ganhou relevância como um teste funcional simples, seguro e aplicável em pacientes com doença cardiovascular, especialmente em cenários onde o teste de caminhada de 6 minutos (TC6) não é viável. Sua utilidade na avaliação da força dos membros inferiores o torna uma ferramenta complementar de crescente interesse, o que pode fortalecer as baterias de avaliação funcional nessa população.

**Objetivo:**

Correlacionar os resultados do TC6 com o STS1 como ferramentas complementares na avaliação funcional de pacientes com doença cardiovascular.

**Materiais e métodos:**

Um estudo descritivo, correlacional e transversal foi conduzido em 45 pacientes internados em um programa de reabilitação cardíaca em uma clínica de atendimento terciário em Cali, Colômbia. O TC6 e o STS1 foram administrados, avaliando variáveis fisiológicas e de desempenho físico. O coeficiente de correlação de Pearson foi utilizado para análise bivariada.

**Resultados:**

A média de idade foi de 62,97 ± 12,58 anos; 64,4% eram mulheres. Doença arterial coronariana foi o diagnóstico mais comum (73,3%). Foi encontrada correlação positiva moderada entre a distância percorrida no TC6 e o número de repetições no STS1 (r = 0,610; p < 0,001). O TC6 gerou maior aumento na frequência cardíaca (final: 112,7 ± 18,1), enquanto o STS1 teve maior impacto na pressão arterial sistólica (PAS final: 146,8 ± 27,9; p < 0,001).

**Discussão:**

Os resultados são consistentes com estudos em outras populações e apoiam o uso do STS1 como uma opção funcional válida.

**Conclusões:**

O STS1 complementa o TC6 na avaliação funcional de pacientes com doença cardiovascular.

## Introduction

Cardiovascular diseases (CVD) encompass a wide range of disorders of the heart and blood vessels, including ischemic heart disease, cerebrovascular diseases, and peripheral arterial disease, among others. The World Health Organization (WHO) reported that in 2019, 17.9 million people died from these conditions, accounting for 32% of all deaths worldwide^1^. In the same year, CVD caused 40.8 million disability-adjusted life years (DALYs) and 36.4 million years of life lost (YLLs) due to premature mortality, according to the Pan American Health Organization (PAHO)^2^. Furthermore, the most recent Global Burden of Disease study reported that ischemic heart disease was the leading cause of death in 2021, with a rate of 108.7 per 100,000 population^3^.

Among the consequences of CVD is impaired physical fitness^4^, which is associated with increased sedentary behavior and physical deconditioning, exacerbating symptoms such as dyspnea and fatigue, as well as the overall clinical features of the disease. Therefore, within cardiac rehabilitation (CR) programs, exercise has been proposed as a cornerstone for restoring functional capacity in patients with CVD; however, a comprehensive assessment is essential, encompassing key components of physical fitness, including muscle strength and cardiorespiratory endurance.

At the baseline assessment upon entry into CR programs, both physiological and physical variables are evaluated. The 6-Minute Walk Test (6MWT) is a safe, low-cost, reliable, and well-tolerated test in patients with CVD^5^,^6^, widely used in clinical settings to estimate functional aerobic capacity. It simultaneously integrates circulatory, ventilatory, and neuromuscular responses, allowing estimation of cardiorespiratory capacity through maximal oxygen consumption (VO2 max), derived from the distance walked. However, the test requires a 30-meter corridor, which makes its implementation challenging in various inpatient and outpatient settings. Therefore, there is a need to identify and validate complementary functional tests that can be applied when the minimum physical requirements for performing the 6MWT are not available, without compromising the quality of functional assessment.

Therefore, various tests have been proposed as part of the complementary assessment of patients with CVD, using simple equipment and limited space, addressing time and space constraints. Among these is the 1-Minute Sit-to-Stand Test (STS1), which assesses lower-extremity muscle strength, a component associated with patients’functional capacity, as its performance relies on simple, everyday movements such as sitting down and standing up from a chair^7^. The STS1 has been used across different health conditions, including chronic obstructive pulmonary disease (COPD)^8^, interstitial lung disease (ILD)^9^, asthma^10^, heart failure^11^, kidney disease^12^, and cancer^13^, among others, demonstrating its utility as an assessment, prognostic, and disease progression indicator.

Considering the above, this study aims to assess the correlation between the 6MWT and the STS1 as a complementary assessment in patients with cardiovascular disease.

## Materials and Methods

A descriptive, correlational, cross-sectional study was conducted in a population of 45 patients aged ≥18 years with a medical diagnosis of cardiovascular disease, including conditions such as coronary artery disease (stable angina, post-revascularization), ischemic heart disease, heart failure, and cardiogenic shock. The study included all participants initiating a cardiac rehabilitation program between February and May 2024 at a quaternary care clinic in Cali, Colombia. The sample size was non-probabilistic and based on convenience, determined by the total number of patients enrolled in the cardiac rehabilitation program during the established data collection period. This approach was adopted because the study was exploratory and conducted in a clinical setting, where access to the population is conditioned by availability and the natural flow of patient admissions within a specific timeframe. Clinical diagnoses were established by the treating cardiologist based on the medical record and current clinical, paraclinical, and imaging criteria, and were coded according to the International Classification of Diseases, Tenth Revision (ICD-10). The disease duration was defined as the time from the initial diagnosis to enrollment in the program and was recorded in months; however, it was excluded from the analysis due to its heterogeneity.

Patients with physical limitations preventing them from performing the tests were excluded, as were those with hemodynamically unstable cardiovascular disease and those with a psychiatric diagnosis.

**Variables**

Sociodemographic variables included age and sex, along with clinical variables specific to the type of diagnosis. In addition, circulatory and ventilatory physiological variables were assessed, including heart rate (HR), systolic blood pressure (SBP), diastolic blood pressure (DBP), mean arterial pressure (MAP), respiratory rate (RR), and oxygen saturation (SpO2). Perceived exertion was also evaluated using the modified Borg scale for fatigue. Additionally, distance walked (meters) and the number of repetitions were analyzed for the 6MWT and the STS1, respectively.

**Procedures**

After providing written informed consent, patients were evaluated in a single session by a physiotherapist specialized in cardiac and pulmonary rehabilitation, who administered a sociodemographic and clinical questionnaire. Upon completion, aerobic capacity was assessed using the 6MWT, in accordance with the American Thoracic Society guidelines^14^. For the STS1, a standard chair 43-46 cm in height with back support was used. Participants were instructed to keep their arms crossed over their chest and to perform as many sit-to-stand repetitions as possible within 1 minute^15^. A 30-minute rest period was allowed between the 6MWT and the STS1 to enable recovery to baseline values.

**Statistical analysis**

Statistical analysis was performed using Stata v.16. After importing the dataset, an exploratory phase was conducted to identify missing data and outliers. When detected, data were verified and re-entered. Subsequently, descriptive analysis was performed. Categorical variables were presented as frequencies and percentages, and continuous variables were assessed for normality using the Shapiro-Wilk test. Given the normal distribution of the data, results are reported as means ± standard deviations (SD).

For the bivariate analysis, Pearson’s correlation coefficient was used to assess the association between cardiovascular and ventilatory physiological variables, as well as perceived exertion, measured at baseline and post-test during the 6MWT and the STS1. For the comparison of mean physiological variables between baseline and post-test, a paired Student’s t-test was used. Statistical significance was set at p < 0.05. All collected data are publicly available for consultation in Mendeley Data^16^.

**Ethical considerations**

This study was conducted in accordance with Resolution 8430 of 1993, which regulates scientific research in Colombia, and was classified as minimal risk. The Declaration of Helsinki was also followed to ensure the protection of participants’ data and to obtain written informed consent, in which the study objectives were explained. Additionally, this study received ethical approval from the Institución Universitaria Escuela Nacional del Deporte (Approval No. 40.07.233). All participants agreed to participate voluntarily and provided written informed consent before study initiation.

## Results

During 2024, a total of 45 patients were enrolled, of whom 64.44% were women. The mean age was 62.97 ± 12.58 years. Regarding clinical characteristics, the most frequent underlying diagnosis was coronary artery disease (73.33%), followed by ischemic heart disease (15.56%), cardiogenic shock (8.89%) and heart failure (2.22%) Table 1.

**Table 1:** Sociodemographic and clinical characteristics of the patients (N = 45)

Variable	(Frequency) % (n)
Sex	
Male	35.56 (16)
Female	64.44 (29)
Age M ± SD	62.97 ± 12.58
Diagnosis	
Coronary Disease	73.33 (33)
Ischemic Heart Disease	15.56 (7)
Cardiogenic Shock	8.89 (4)
Heart failure	2.22 (1)

M: Mean; SD: Standard deviation

Table 2 shows the cardiovascular and ventilatory responses to both tests. The 6MWT elicited a greater increase in final heart rate (112.73 ± 18.08) compared with the STS1 (94.60 ± 14.55). In contrast, the STS1 had a greater effect on SBP and DBP and, consequently, on MAP (p < 0.001), with an increase of 5.9 mmHg at the end of the test, whereas the 6MWT increased it by only 0.12 mmHg.

**Table 2:** Baseline and post-test physiological responses for the 6MWT and the STS1

Variable	6MWT			STS1			p-value *
	Baseline	Post-test	p-value	Baseline	Post-test	p-value	
HR	80.67 ± 12.43	112.73 ± 18.08	<0.001	79.89 ± 12.31	94.60 ± 14.55	<0.001	<0.001
SBP	130.89 ± 19.07	131.42 ± 21.85	0.835	137.00 ± 23.06	146.75 ± 27.88	<0.001	<0.001
DBP	78.00 ± 13.08	77.91 ± 11.43	0.968	79.93 ± 13.15	83.91± 15.25	0.010	0.006
MAP	95.62 ± 13.70	95.74 ± 12.74	0.954	98.95 ± 14.58	104.85 ± 16.81	<0.001	<0.001
SpO_2_	96.11 ± 2.55	93.89 ± 4.11	<0.001	95.89 ± 4.66	95.22 ± 4.59	0.468	0.005
RR	20.84 ± 4.77	28.31 ± 6.95	<0.001	20.98 ± 5.24	24.27 ± 5.23	<0.001	<0.001

6MWT: 6-Minute Walk Test; STS1: 1-Minute Sit-to-Stand Test; HR: heart rate; SBP: systolic blood pressure; DBP: diastolic blood pressure; MAP: mean arterial pressure; SpO2: oxygen saturation; RR: respiratory rate. * p-value for the comparison of mean post-test values between the 6MWT and the STS1. Student’s t-test: values are presented as mean ± standard deviation (SD).

Regarding physiological variables at baseline for the 6MWT and the STS1, strong positive correlations were observed for HR (r = 0.952; p< 0.001), DBP (r = 0.879; p< 0.001), MAP (r = 0.810; p< 0.001), and RR (r = 0.965; p< 0.001). Moderate positive correlations were also found for SBP (r = 0.674; p< 0.001) and SpO2 (r = 0.695; p< 0.001), all of which were statistically significant. At post-test assessment for both tests, a strong positive correlation was observed for SpO₂ (r = 0.760; p< 0.001), while moderate positive correlations were found for HR (r = 0.598; p< 0.001), SBP (r = 0.617; p< 0.001), DBP (r = 0.417; p = 0.001), MAP (r = 0.670; p< 0.001), and RR (r = 0.669; p< 0.001), all of which were statistically significant Table 3.

A moderate positive correlation was observed between the number of repetitions performed in the STS1 and the distance covered in the 6MWT, which was statistically significant (p< 0.001).

**Table 3:** Correlation between cardiovascular and ventilatory physiological variables and perceived exertion at baseline and post-test for the 6MWT and the STS1

Variable	HR STS1	SBP STS1	DBP STS1	MAP STS1	SpO_2_ STS1	RR STS1	Fatigue STS1	STS1* (Repetitions)
Baseline								
HR 6MWT	0.952**							
SBP 6MWT		0.674**						
DBP 6MWT			0.879**					
MAP 6MWT				0.815**				
SpO_2_ 6MWT					0.473*			
RR 6MWT						0.965**		
Fatigue 6MWT							0.182	
Post-test								
HR 6MWT	0.598**							
SBP 6MWT		0.617**						
DBP 6MWT			0.471*					
MAP 6MWT				0.670**				
SpO_2_ 6MWT					0.760**			
RR 6MWT						0.669**		
Fatigue 6MWT							0.034	
6MWT (Distance)								0.616**

HR: heart rate; SBP: systolic blood pressure; DBP: diastolic blood pressure; MAP: mean arterial pressure; SpO2: oxygen saturation; RR: respiratory rate; 6MWT: 6-Minute Walk Test; STS1: 1-Minute Sit-to-Stand Test. Pearson correlation; * p < 0.01, ** p <0.001.

## Discussion

CVDs significantly impair patients’ physical function, reducing cardiorespiratory endurance and muscle strength, which in turn affects their daily activities. Therefore, clinical assessment and functional capacity stratification are essential for prescribing appropriate rehabilitation programs.

The 6MWT is widely used to assess functional capacity across various conditions, demonstrating good performance in evaluating exercise capacity. However, space constraints in some clinical settings necessitate the identification of alternative tests. In this context, the STS1, initially used in healthy older adults, has emerged as a viable option for assessing functional capacity in patients with chronic diseases^17^, as it is easy to administer, requires minimal space, and provides information on lower-extremity muscle strength^7^. This study examines the correlation between the 6MWT and the STS1 as a complementary tool for evaluating functional capacity in patients with cardiovascular disease.

Regarding sociodemographic characteristics, a higher proportion of participants were women, with a mean age of 62.9 ± 12.58 years. These results are consistent with similar studies in terms of age^18^,^19^, but differ regarding sex, as participants in those studies were predominantly male. Additionally, some studies have focused on a single group within cardiovascular diseases, as they did not include multiple conditions within this broad category^19^,^20^, whereas the present study includes coronary artery disease, ischemic heart disease, cardiogenic shock, and heart failure.

The STS1 and the 6MWT require different movement patterns to be performed; however, similarities were observed in physiological responses to physical exertion, including both cardiovascular and ventilatory variables. Heart rate was higher at the end of the 6MWT compared with the STS1, consistent with findings reported in other studies across different patient populations (Ozel et al.^20^, Meriem et al.^21^, Wang et al.^19^, and Watson et al.^22^). In patients with cardiovascular disease, the greater increase in heart rate during the 6MWT is related to the duration and sustained nature of the effort, as it requires a continuous supply of oxygen to the muscles, increasing cardiac workload and cardiac output to pump blood and meet the metabolic demands of continuous aerobic exercise. In contrast, during the STS1, physical effort is intermittent, and oxygen demand decreases between repetitions, allowing partial recovery of heart rate^20^,^21^.

A significant increase in SBP was observed at the end of both tests, with a slightly greater increase in SBP during the STS1, which also influenced MAP. Nevertheless, findings in the literature are inconsistent regarding blood pressure responses, as some studies^19^,^20^ have reported a greater increase in SBP with the STS1, whereas others^23^-^25^ have reported similar responses for both tests.

The STS1 requires rapid movements involving quick changes in posture, which reduce central blood volume. This leads to rapid activation of the autonomic nervous system, particularly the sympathetic nervous system, increasing heart rate, peripheral vascular resistance, and consequently SBP as an orthostatic response^26^ to maintain cerebral and coronary blood flow. However, in patients with cardiovascular disease, compensatory mechanisms may be impaired due to prior cardiac surgery, medication use, or autonomic dysfunction^27^.

The findings of this study regarding SBP during the STS1 are related to postural changes, which impose an immediate cardiovascular demand, in contrast to the 6MWT, which elicits a slower but sustained response over time, with more stable SBP behavior during the test. Therefore, particular caution is warranted in postoperative patients with cardiovascular disease to avoid adverse outcomes during functional assessment, as blood pressure regulatory mechanisms may be impaired. Additionally, the STS1 involves rapid and physically demanding postural changes in this patient population, requiring adequate cardiovascular adaptation.

Regarding the correlation between distance covered in the 6MWT and the number of repetitions in the STS1, a strong positive correlation was observed (r = 0.610; p< 0.05). These findings are supported by several studies, such as that of Ozel et al.^19^, which reported a strong association between these variables (r = 0.809; p< 0.001) in patients with atrial fibrillation. Similarly, Tanriverdi et al.^23^ reported a moderate-to-strong correlation (r = 0.612; p< 0.001) in patients with chronic heart failure. Wang et al.^19^ also found a moderate correlation (r = 0.55; p< 0.001) between both tests in patients with coronary artery disease. In that study, patients with stable coronary artery disease, post-acute myocardial infarction, or following percutaneous coronary intervention were included, demonstrating that the STS1 shows convergent validity with the 6MWT and can discriminate cardiovascular risk levels. In addition, they reported high test-retest reliability (ICC = 0.96) and an area under the curve (AUC) of 0.80 for the STS1, with good sensitivity and specificity for predicting a high risk of cardiovascular events.

In patients with heart failure, Tanriverdi et al.^23^ evaluated the validity and reliability of the STS1 in individuals with chronic heart failure, reporting excellent test-retest reliability (ICC = 0.932) and a significant correlation with the 6MWT, functional class, muscle strength, and pulmonary function. Their findings indicate that the STS1 elicits physiological demands similar to those of the 6MWT, with the advantage of requiring less space and time^23^. Triangto et al.^28^ demonstrated that the 30-second sit-to-stand test shows a strong correlation with the 6MWT (r = 0.629; p< 0.001) in patients with chronic heart failure with systolic dysfunction, reinforcing its utility as a marker of cardiorespiratory fitness. These results are consistent with the findings of the present study.

Moreover, this correlation has been widely studied in patients with COPD^29^-^33^, supporting the use of the STS1 as a complementary tool for estimating functional capacity. Although the pattern of lower-extremity muscle activation may be similar between both tests, it is important to recognize that the underlying energy systems differ, with the 6MWT being predominantly aerobic and the STS1 involving greater anaerobic contribution, which may influence outcomes such as oxygen consumption (VO2). However, both tests integrate cardiovascular, respiratory, and musculoskeletal responses, and their combined use has proven relevant across diverse clinical settings. Therefore, the STS1 does not replace the 6MWT but may serve as a valid alternative when the latter is not feasible, provided its application and interpretation account for these physiological differences.

This study has several strengths, including the combined analysis of the STS1 and the 6MWT in a real-world clinical setting, providing evidence applicable to cardiovascular rehabilitation programs. It also highlights the assessment of key physiological variables and the inclusion of diverse cardiovascular diagnoses, allowing exploration of the utility of the STS1 across a broad spectrum of patients. However, the findings should be interpreted within the context of these considerations, and future studies with larger sample sizes and more homogeneous subgroups are needed to enhance generalizability.

Among the limitations of this study are the small sample size and the absence of maximal exercise testing, such as cardiopulmonary exercise testing (CPET), for direct validation of functional capacity. Additionally, disease duration and the presence of comorbidities were not included in the analysis. However, the latter limitation does not affect the results obtained, as the exclusion criteria accounted for clinical conditions that could alter physiological responses to physical testing, such as unstable cardiovascular disease and musculoskeletal or psychiatric disorders.

## Conclusions

The findings of this study demonstrate that the STS1 shows a significant positive correlation with the 6MWT in patients with cardiovascular disease, supporting its use as a complementary tool for functional assessment. Although both tests evaluate different components of functional capacity, the similar physiological responses—characterized by greater cardiovascular demands in the 6MWT and greater hemodynamic responses in the STS1—indicate that the STS1 may be a valid option in clinical settings where space or resources are limited.

This correlation reflects the integration of muscle strength and aerobic endurance, both essential components in cardiac rehabilitation. The STS1, by requiring less space and time, offers practical advantages without compromising the quality of functional assessment.

Despite the limitations related to sample size and the heterogeneity of diagnoses, this study provides relevant evidence that reinforces the role of the STS1 in clinical practice. A key strength is the comparative physiological analysis between both tests, which allows for a more comprehensive clinical interpretation of their applicability. These findings underscore the need for further research with larger and more homogeneous samples to strengthen the external validity of these results.
